# Combination of Microstereolithography and Electrospinning to Produce Membranes Equipped with Niches for Corneal Regeneration

**DOI:** 10.3791/51826

**Published:** 2014-09-12

**Authors:** Ílida Ortega, Farshid Sefat, Pallavi Deshpande, Thomas Paterson, Charanya Ramachandran, Anthony J. Ryan, Sheila MacNeil, Frederik Claeyssens

**Affiliations:** ^1^Department of Materials Science and Engineering, University of Sheffield; ^2^Department of Chemistry, University of Sheffield; ^3^L. V. Prasad Eye Institute

**Keywords:** Bioengineering, Issue 91, electrospinning, microstereolithography, stem cell niche, storage, limbal explants

## Abstract

Corneal problems affect millions of people worldwide reducing their quality of life significantly. Corneal disease can be caused by illnesses such as Aniridia or Steven Johnson Syndrome as well as by external factors such as chemical burns or radiation. Current treatments are (i) the use of corneal grafts and (ii) the use of stem cell expanded in the laboratory and delivered on carriers (*e.g.*, amniotic membrane); these treatments are relatively successful but unfortunately they can fail after 3-5 years. There is a need to design and manufacture new corneal biomaterial devices able to mimic in detail the physiological environment where stem cells reside in the cornea. Limbal stem cells are located in the limbus (circular area between cornea and sclera) in specific niches known as the Palisades of Vogt. In this work we have developed a new platform technology which combines two cutting-edge manufacturing techniques (microstereolithography and electrospinning) for the fabrication of corneal membranes that mimic to a certain extent the limbus. Our membranes contain artificial micropockets which aim to provide cells with protection as the Palisades of Vogt do in the eye.

**Figure:**
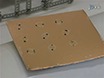


## Introduction

The cornea, the avascular central outer most tissue of the eye, is one of the most important tissues involved in vision^1^. There are several types of cells that maintain the function of the cornea. The top outermost layer of the cornea comprises of epithelial cells which can be about 5-7 layers in thickness^2^. This layer prevents bacterial invasion into the cornea^3^ and allows entry of oxygen^4^. It has been reported that the stem cells of the corneal epithelium lie in niches or crypts (with sizes of 120-150 µm) at the peripheral region of the cornea known as the limbus^5,6^. As the stem cells divide, the daughter cells also known as transient amplifying cells travel out of the niches and as division continues the cells move centripetally inwards and upwards resulting in terminally differentiated cells at the central corneal region^7,8^. These cells are routinely wiped away with the blink of the eye exposing newer cells underneath^9^.

In addition to being the location of the epithelial stem cells, the limbus also plays a role in keeping the vascularized conjunctiva away from the cornea region^10^. Damage to the limbus could be caused by thermal/chemical burns, radiation and also genetic diseases^10^. When this happens, the limbus barrier is broken down allowing the conjunctival cells to move onto the cornea, vascularizing the region, causing pain and blindness in some cases. The condition is known as limbal stem cell deficiency (LSCD)^10^.

Different natural substrates have been reported as possible stem cell carriers for aiding in corneal regeneration. For example, collagen-based membranes were used by Dravida *et al*.^11 ^and Rama and coworkers^12^ reported the use of fibrin in a study with 112 patients. At present however the most commonly used method of treatment is to use human amniotic membrane from a tissue bank and culture limbal epithelial cells on its surface^13,14^. Once a monolayer has formed, the amniotic membrane is glued cell-side up onto the damaged cornea which has all the conjunctival cells and scar tissue surgically removed from it prior to this cell transplantation^14^. The amniotic membrane degrades within weeks to months leaving the epithelial cells attached to the denuded area to regenerate the epithelium^15,16^. This technique has been successful in restoring vision however there are still a few practical issues which restrict its widespread uptake clinically. As the amniotic membrane is human tissue it needs to undergo screening using good tissue banking procedures before being used for cell transplantation on patients. This screening only lowers the risk of transmission of diseases but cannot completely eliminate it^17^. In addition to this there have been reports of variability in the performance of the amniotic membrane due to inter donor variation^18,19^ and different processing methods^19,20^. Alongside the small risk of disease transmission there is the requirement for surgical centers to have access to well-run tissue banks, not available to all.

Although the amniotic membrane is relatively successful, there is a need for the development of new synthetic biodegradable cell carrier alternatives for the treatment of corneal disease. Synthetic carriers would overcome the need for banking procedures as well as eliminating the small risk of disease transmission and inter-donor variability. In this sense, materials such as polyethylene glycol^21,22^ and PLGA^23,24^ have been studied.

In developing a synthetic alternative to the human amniotic membrane there is also the possibility to design into it desirable features to hopefully help the survival of the cultured cells. The inclusion of microfeatures within biomaterial devices for the specific control of cell behavior is an emerging area of interest. Many authors have reported work towards the development of artificial stem cells niches^25-30^. This group has recently reported the creation of a microfabricated PEGDA fibronectin-biofunctionalized artificial limbus for the delivery of limbal epithelial cells^22^ and a methodology for the fabrication of electrospun biodegradable membranes containing microfabricated pockets for the support of limbal epithelial cells^31^.

The aim of this work is to develop a new manufacturing technology for the development of biomaterial devices containing microfeatures which mimic to an extent the microenvironments in which stem cells reside in the body. We have developed a technique which combines microstereolithography and electrospinning that allows the fabrication of biodegradable microstructured membranes that show great potential for tissue regeneration applications.

It is important to notice that although in this work this technique has been applied to the fabrication of rings for corneal regeneration, the technology can be applied to the fabrication of devices for the regeneration of a broad range of epithelial tissues, *e.g.*, skin, oral mucosa, intestine, respiratory, and bladder epithelia. Specifically, in this study we have developed a synthetic biodegradable membrane which functions in a similar manner to the amniotic membrane to deliver cells to the cornea. This membrane contains micropockets of around 300 µm (larger than the limbal crypts of the Pallisades of Vogt (around 150 µm)). Finally, we have established a packaging protocol which allows these membranes to be stored at -20 °C for more than 6 months without showing any signs of breakdown.

## Protocol

Ethics Statement: The eyes used in this study were used according to the Declaration on Openness on Animal Research:

### 1. Fabrication of PLGA Biodegradable Membranes Equipped with Micropockets

1. The ring scaffolds were created by a combination of microstereolithography and electrospinning techniques^31^. In essence, the process can be summarized in 2 parts (i) creation of PEGDA templates by microstereolithography and (ii) electrospinning onto the templates for reproduction of the underlying PEGDA structure (in this case a microfabricated ring). These two steps are described in detail below (**Figures 1** and **2**).
  1. Fabrication of PEGDA templates by microstereolithography (microSL)
    1. Fabricate the rings using a 2 layer model, with the first layer (L1) being the base of the structure and the second layer (L2) presenting 6 micropockets with horseshoe morphologies in a range of sizes from 300-500 µm. The fabrication of PEGDA rings was recently described by this group^22^.
      1. Create L1 by drawing a 1.2 cm black circle using any suitable drawing program.
      2. Create L2 in the same way but include 8 white 0.5 x 0.35 mm horseshoe-shaped structures. Distribute the small white horseshoe shapes within the black circle structure.
      3. Save L1 and L2 in JPEG format.
    2. In a dark glass vial, mix polyethylene glycol diacrylate (PEGDA, Mn = 250 g/mol) with 1% w/w Camphorquinone, a photoinitiator, on a magnetic stirrer for 20 min.
    3. Expand the laser beam of the microstereolithography set-up using a telescopic lens arrangement and then project it onto a computer programmable digital multimirror device (UV-enabled DMD starter kit). NOTE: The DMD reflects the image (in this case a ring) onto a mirror via a 10 cm focal length tube lens. The image is then directed by the silver-coated mirror into a vial containing the photocurable polymer (PEGDA).
    4. Adjust and carefully clean the optics of the microstereolithography set up.
    5. Put 300 µl of the PEGDA mixture into a well of a 12-well tissue culture plate. Make sure the wells are pre-coated with Teflon or other non-stick material for easy removal of the structure after curing.
    6. Switch on the blue laser (MBL-III 473 nm; 150 mW) and upload L1 into the ALP3-basic software previously installed on the PC. Irradiate the first layer for 60 sec. NOTE: ALP3-basic is a USB interface that provides the link between the PC and the Digital micromirror device.
    7. Add to the well 250 µl more of the PEGDA, upload L2 as described above and irradiate L2 for 60 sec.
    8. Remove the uncured polymer and wash the ring with isopropanol O/N.
  2. Fabrication of Biodegradable PLGA membranes using electrospinning NOTE: The PEGDA rings were used as templates over which PLGA was electrospun with the PLGA reproducing the underlying topography as it is spun over these templates. After spinning, the sheet of PLGA polymer was peeled from the collector. The final PLGA membrane did not contain the PEGDA rings which were left attached to the metallic collector.
    1. Distribute the PEGDA rings on an electroplated aluminum sheet (12 cm x 20 cm) for creating a static electrospinning collector.
    2. Attach the rings using conductive carbon tape. NOTE: These rings once formed can be re-used as templates for electrospinning.
    3. Prepare the polymer solution for spinning. Dissolve PLGA (50/50 DL-lactide (52 mol%): glycolide (48 mol%), 44 kg/mol) in dichloromethane (DCM) at 20% w/w concentration.
    4. Stir O/N before use.
    5. Place 4 insulin syringes (blunt ended, 0.8 cm inner diameter needles) on a syringe pump. NOTE: Four syringes ensured more rapid electrospinning than working with a single syringe.
    6. Load 2.5 ml of PLGA solution in each syringe.
    7. Electrospin using a 30 µl/min flow rate and voltages ranging from 12 to 15 kV. Leave a distance between the needles and the collector of 15 cm.
    8. Electrospin for 1 hr and 30 min and finally carefully peel the PLGA electrospun sheet from the collector supporting the PEGDA rings.
    9. Cut the electrospun scaffolds into 22 mm diameter circles using a circular hole punch and leaving the ring structure positioned in the center.

### 2. Long-term Storage of PLGA Microfabricated Membranes

Note: The PLGA rings were fabricated and sterilized by accredited external companies; samples were irradiated at an external dose range of 25-40 KGy.

1. Mount the membrane in a small container (Plastic petri dish) and place it inside a medical grade bag.
2. Use filter paper to create small filter bags for the desiccants. To create the bags, fold the filter paper (125 mm diameter) and cut the resulting semicircle in half; then seal the ends together using tape.
  1. Fill three filter paper bags with 1 g of silica orange, cobalt (II) chloride, and copper (II) sulfate, respectively.
  2. Put the three bags of desiccant inside the medical grade bag along with the electrospun membrane.
3. Add to the bag a commercially available six spot humidity indicator card to detect any moisture accumulation during the storage period.
4. Use a vacuum heat seal machine to vacuum and seal the bag.
5. Send the ring membranes to an external company for γ-irradiation.
6. Store γ-irradiated PLGA membranes at a wide range of temperatures from -20 °C to +37 °C in a moist environment within an incubator containing 5% CO_2_.
7. Post storage, examine the humidity indicator to confirm that the level of humidity is beneath 30%.
8. Use Scanning Electron Microscopy (SEM) to assess fiber integrity.

### 3. Isolation of Limbal Explants

Rabbit limbal explants were isolated from rabbit eyes (obtained from a farm where rabbits are bred for consumption).

1. Disinfect the rabbit eyes using antiseptic solution (3%).
2. Clean the eyes by removing any excess tissue surrounding the cornea.
3. Separate the limbal region (identified as a thin circular area between the cornea (transparent) and the sclera (white)) from the rest of the cornea using a dissection microscope.
4. Cut into segments (around 1.5 cm long) under the dissection microscope.
5. Disinfect the limbal segments in 1.5% antiseptic solution for 1 min.
6. Cut the limbal segments into small pieces (100-500 µm) with a scalpel blade.
7. Store the small pieces of tissue in culture media (DMEM+Glutamax: Ham´s F12 (1:1), 10% fetal bovine serum, 1 U/ml penicillin, 100 mg/ml streptomycin, 2.5 µg/ml amphotericin, 10 ng/ml of EGF, and 5 µg/ml of insulin) at 37 °C and 5% CO_2 _until use (no more than 60 min).

### 4. Outgrowth of Cells from Limbal Explants

Rabbit limbal explants were placed on both freshly spun microfabricated membranes and membranes which were vacuum-packed and stored for 6 months at -20 °C.

1. Coat the ring scaffolds with 15 µl of fibrin glue (1:1 mixture of fibrinogen from human plasma at a concentration of 18.75 mg/ml and thrombin from human plasma at a concentration of 2.5 U/ml) using a cell scraper for distributing the fibrin evenly.
2. Place the tissue explants directly on the PLGA micropockets using a 25 G needle and a dissecting microscope.
3. Add cell culture media very gently to avoid detaching the explants.
  1. Change media every 3 days (media recipe described in section 3.7) and keep in culture for 2 weeks at in a humidified incubator at 37 °C and 5% CO_2_.
4. Fix the samples with 3.7% buffered formaldehyde for 10 min followed by 3 washes with PBS.
5. Counterstain by incubation in 1 µg/ml of 4',6-diamidino-2-phenylindole (DAPI) or propidium iodide (PI) for 10 min at RT.
6. Wash 3 times in PBS and store the samples covered with foil.
7. Image the samples using a fluorescence microscope at wavelengths of 543 nm and 800 nm (two-photon).

### 5. Setting-up Rabbit Wounded Cornea 3D Models

1. Clean and disinfect the rabbit eyes as described above.
2. Wound the rabbit eyes by immersing them in 0.14% ammonium hydroxide for 5 min.
3. Rinse the eyes in PBS and scrape away the epithelium with a sclerotome knife.
4. Cut and isolate the cornea-scleral button removing any remaining tissue.
5. Place the corneas epithelial side down onto a sterile cup and fill with 0.5% agar made in DMEM.
6. Once set, put the corneas epithelial side up, in small petri dishes and add culture media (recipe described above) up to the limbal area. NOTE: Do not cover the whole cornea; keep the organ culture at the air-liquid interface.

### 6. Isolation of Limbal Explants and Inclusion of Ring Scaffolds in Rabbit Cornea 3D Models

1. Coat the ring membranes with 15 µl of fibrin glue (1:1 mixture of fibrinogen at a concentration of 18.75 mg/ml and thrombin at a concentration of 2.5 U/ml).Use a cell scraper as described above.
2. Place the tissue explants directly on the PLGA micropockets using a 25 G needle and a dissecting microscope.
3. Place the rings with the tissue explants on the denuded corneas with the explants facing up and at air-liquid interface conditions. (These conditions have been previously described^22^).
4. Maintain the organ culture models for 4 weeks in a humidified incubator at 37 °C and 5% CO_2_ changing the media every 2 days.

### 7. Assessment of Corneal Regeneration and Stem Cell Maintenance

1. After 4 weeks, fix the corneas using 3.7% formaldehyde.
2. Process the corneas for conventional histology to produce 6 µm paraffin sections.
3. Stain with hematoxylin and eosin (H&E).
4. For immunocytochemistry, dewax the sections in xylene (3min) and rehydrate in 100% ethanol (1 min), 70% ethanol (1 min), and distilled water (2 min).
5. Delineate the sections using a Dako pen to delimit small areas and avoid excessive use of antibody.
  1. Treat the delineated areas with 0.05% trypsin (100 µl) for 20 min (37 °C).
6. Wash thoroughly with PBS and add 100 µl of 10% goat’s serum (blocking) for 1 hr.
7. Incubate the samples with 100 µl of mouse monoclonal antibody cytokeratin 3 (CK3) and 100 µl of p63 in 1% goat’s serum O/N at 4 °C.
8. Wash with PBS and treat with 100 µl of biotinylated secondary anti-mouse antibody (1:1,000 in 1% goat serum) for 1 hr at RT.
9. Add 100 µl of FITC-streptavidin (1:100 in 1% goat serum) for 30 min at RT.
10. Treat the samples with DAPI as described above.
11. Image the samples using a fluorescence microscope at wavelengths of 800 nm (two-photon) and 488 nm.

## Representative Results

Electrospun microfabricated rings were manufactured using a combination of microstereolithography and electrospinning (**Figures 1** and **2**). PEGDA rings of different sizes were fabricated using microstereolithography (**Figure 3**); this technique allows the fabrication structures in the order of cm and the simultaneous incorporation of microfeatures. In this case, rings of diameters ranging from 1.2-1.6 cm containing micropockets of 350-500 µm were fabricated (**Figure 4**).

In terms of producing, sterilizing and packaging of materials for future clinical use it was found that vacuum packing in medical grade bags significantly improved the ability to achieve long term storage of PLGA membranes (**Figure 5**); the use of a medical grade bag (PET/Foil/LDPE) with thickness of 0.12 mm allowed us to achieve a longer shelf life. This was investigated by sending membranes to our collaborators in India and membranes were stored for a period of months at -20 °C, at RT and at 37 °C in deliberately moist conditions (a moist incubator). **Figure 5** shows that using the deliberately provocative conditions of storage at 37 °C under moist conditions, membranes were only stable for approximately 1 month under non-vacuum packed conditions, but achieved 3 months storage under vacuum packed conditions (**Figure 5** and **Table 1**).

Table 1 demonstrates the improvement in storage conditions that can be achieved even under conditions selected to be conducive to water uptake and fiber swelling if one pays attention to the choice of bag used.

The rings supported cell outgrowth from limbal explants in different conditions (i) rings freshly made and (ii) rings stored for 6 months (**Figure 6**). Cell transfer was achieved after 4 weeks when placing the PLGA membranes on 3D wounded models. Cells grew out from the tissue explants placed on the membranes creating a new epithelium on the previously denuded corneas (**Figure 7**). Positive (corneas without any treatment) and negative controls (wounded corneas) were also maintained in culture for the same periods of time. The negative controls confirmed the lack of formation of a new epithelium in the absence of any added cells. Immunocytochemistry demonstrated that the cells growing out from the explants were corneal epithelial cells since they were positive for the corneal differentiation marker CK3 (**Figure 7E**).


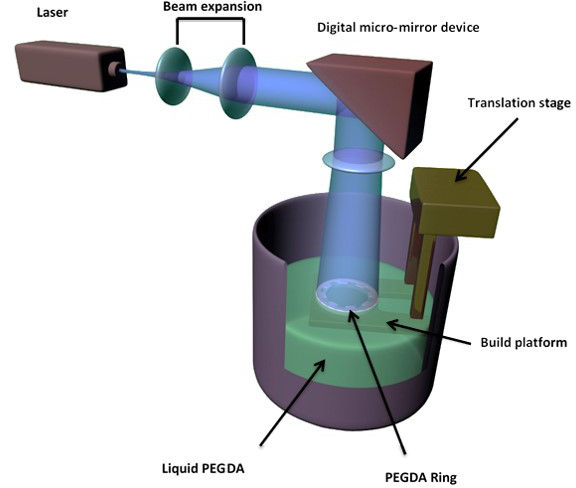
 **Figure 1.** **Schematic of microstereolithography set up for the creation of PEGDA rings.**


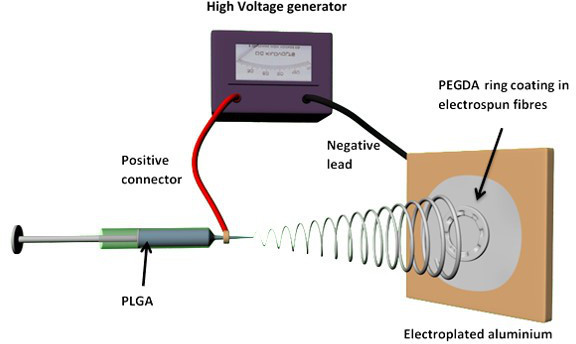
 **Figure 2.** **Schematic of electrospinning process using PEGDA microfabricated rings as a templates.**


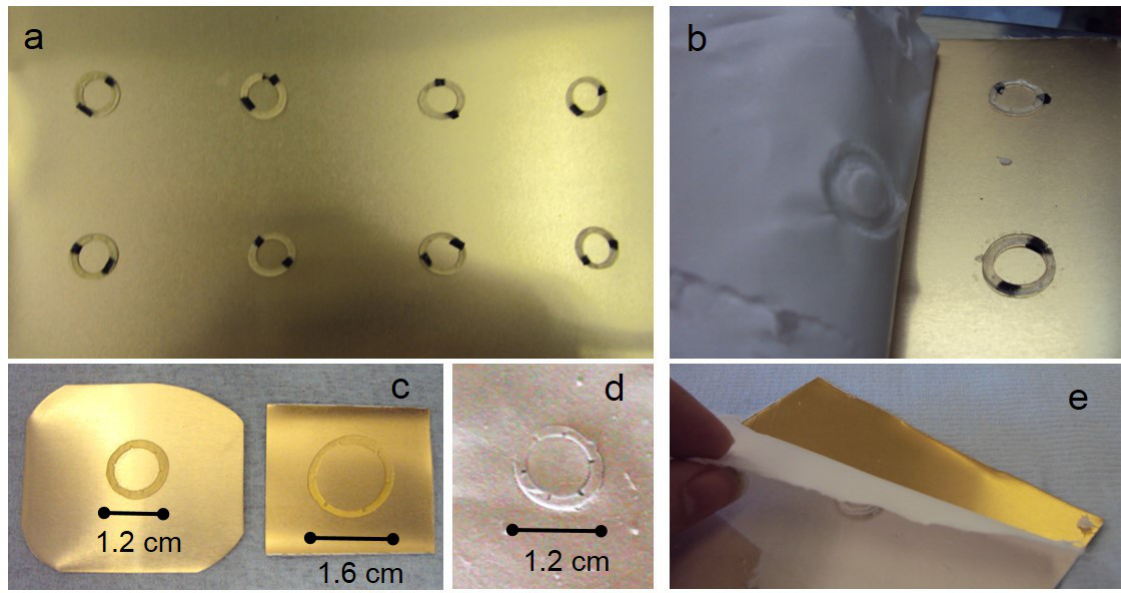
**Figure 3. (A) **shows an example of a static collector (electroplated aluminum sheet with PEGDA rings) for the spinning of microfabricated PLGA membranes. **(B)** and **(E)** show different electrospun mats being peeled from static collectors. **(C)** shows PEGDA templates of different sizes highlighting the versatility of using microstereolithography for the fabrication of the underlying surface. **(D)** shows a PLGA microfabricated replica. Please click here to view a larger version of this figure.


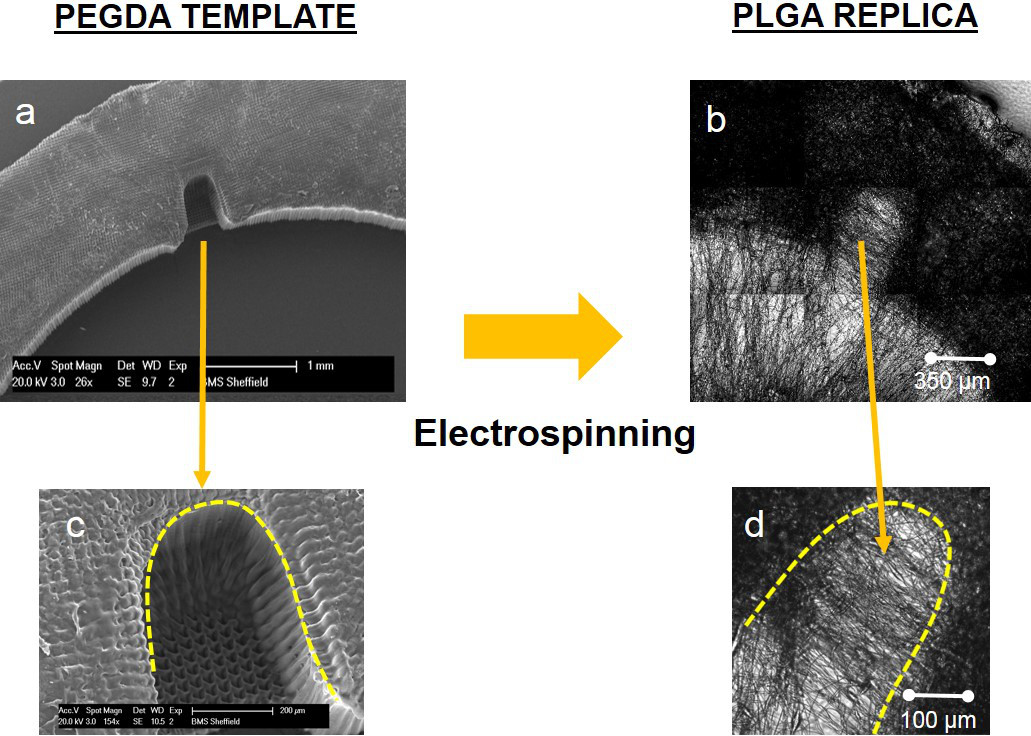
**Figure 4.** SEM image of a PEGDA ring with a horseshoe microfeature **(A)**; high magnification SEM image of a microfabricated pocket **(B)**. Phase contrast image of a PLGA ring with a horseshoe microfeature **(C)**; high magnification phase contrast image of a microfabricated pocket **(D)**. Please click here to view a larger version of this figure.


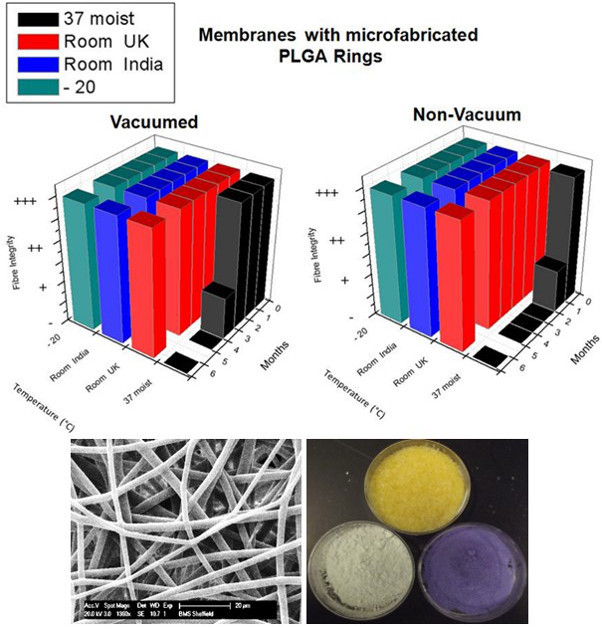
**Figure 5.Effect of temperature and time on storage of vacuum and non-vacuum packed PLGA (50/50) membranes (44 kg/mol) with micro-fabricated rings over 6 months.** Membrane integrity was scored as fully intact fibers (+++), some fiber swelling (++), fiber merging (+) or no intact fibers (-). SEM images and three desiccants (silica orange, cobalt (II) chloride, and copper (II) sulfate) show no changes in fiber integrity or humidity. Please click here to view a larger version of this figure.


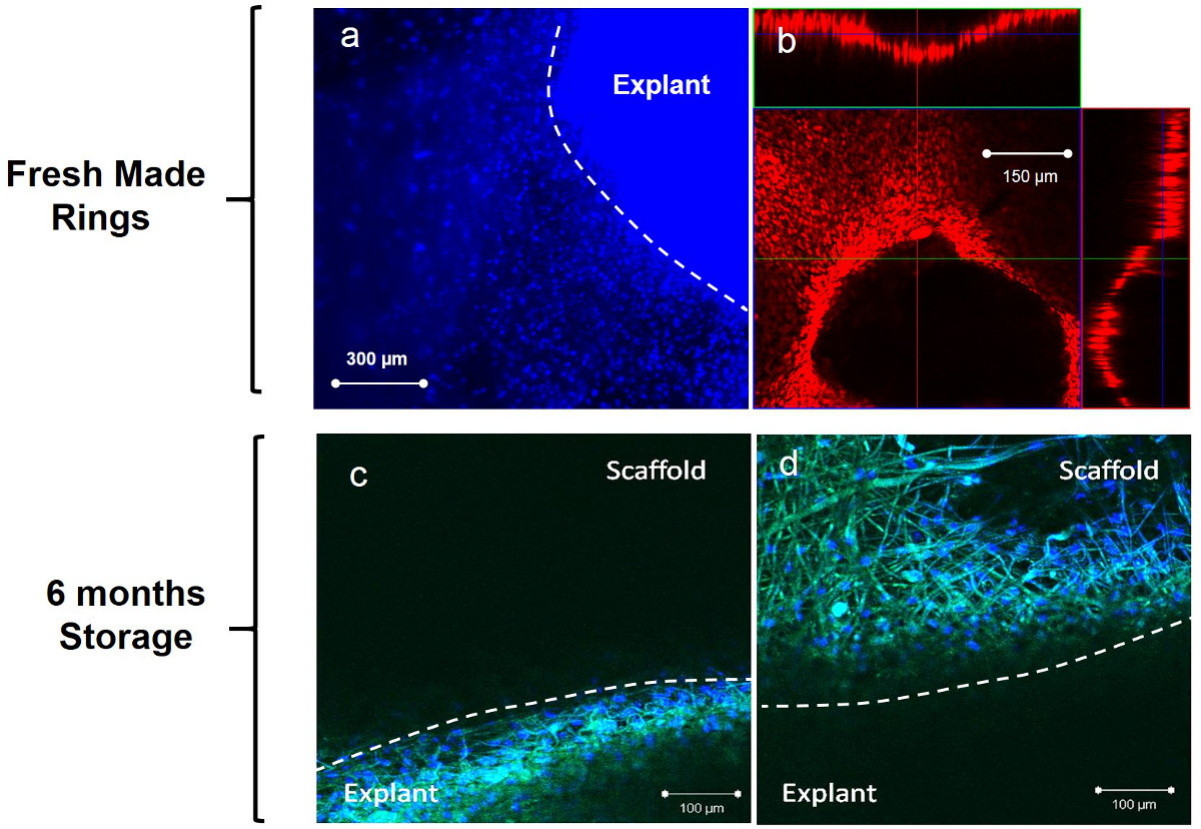
**Figure 6.Fluorescence images showing outgrowth of LEC from limbal explants on freshly made biodegradable PLGA rings (A, B) and on rings after 6 months storage at -20 °C (C, D). **Images **(A)** and **(B)** correspond to cells stained with DAPI (blue) and propidium iodide (red) respectively. Image b is an orthogonal view from a confocal z-stack of an explant placed on a microfabricated pocket. Images **(C)** and** (D) **show positive staining for p63 (green). Please click here to view a larger version of this figure.


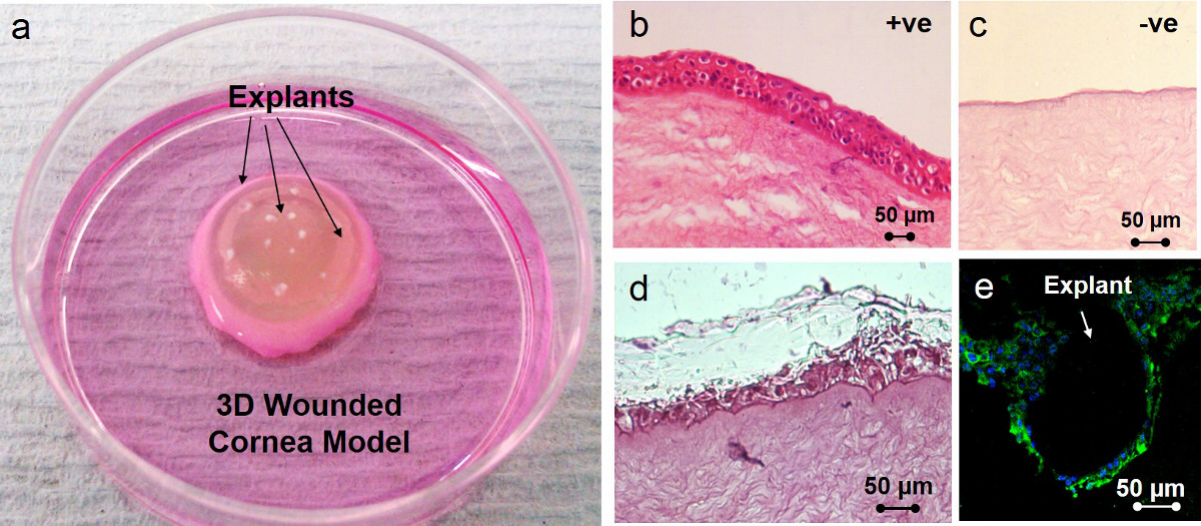
**Figure 7. (A)** shows a rabbit wounded cornea model with a ring scaffold and tissue explants located on the scaffold which was previously coated with fibrin glue. **(B)** and **(C)** are positive and negative controls; the positive control is a fresh rabbit cornea and the negative control a cornea where the epithelium was deliberately removed (the negative control was also cultured for 4 weeks). **(D)** is a H&E image of a tissue engineered cornea after 4 weeks in culture; the figure shows the new multi-layered epithelium formed by the cells coming out from the explants placed on the niches. **(E)** is an immunocytochemistry image showing cell outgrowth from a limbal explant; nuclei are stained with DAPI (blue) and the cells shows positive staining for cytokeratin 3, a corneal differentiation marker (green). Please click here to view a larger version of this figure.

**Table:** 

PLGA (50/50)	Fiber integrity for membranes stored at 37 °C moist
Day 0	Month 1	Month 2	Month 3	Month 4	Month 5	Month 6
Non-Vacuum Packed	+++	-	-	-	-	-	-
Vacuumed (Bag A) (PE, PA Composite) Thickness: 0.14 mm	+++	+	-	-	-	-	-
Vacuumed (Bag B) (PET / Foil / LDPE) Thickness: 0.75 mm	+++	+++	+++	+	-	-	-

**Table 1.Effect of vacuum and different storage bags on integrity of PLGA (50/50) membranes (44 kg/mol) examined over 6 months of storage. **Membrane integrity was scored as fully intact fibers (+++), some fiber swelling (++), fiber merging (+) or no intact fibers (-).

## Discussion

This study describes (a) a technique for the fabrication of electrospun membranes containing microfeatures within them and (b) how to prepare such membranes for clinical use by vacuum packing, gamma irradiation and then storage prior to use. In this particular application we have developed PLGA membranes containing micropockets which mimic the physical features of the limbal stem cell niches. The aims of this study are (i) to describe methods to provide readers with the knowledge needed to design and fabricate scaffolds containing microfeatures for research into the contribution of stem cell niches to tissue regeneration and (ii) to provide the reader with a better understanding of how to store electrospun scaffolds for long periods of time.

In terms of clinical application, the storage of the ring membranes is of paramount importance. In this work, the degradation of the ring was studied over a period of 6 months. Degradation of the membranes is driven by hydrolysis so by simply keeping the membranes moisture-free the process is halted. Blackwood *et al. *reported that by varying the ratio of PLA to PGA, the degradation of the membrane changes^32^. This study also showed that by increasing the amount of PGA, the degradation rate of electrospun membranes increased *in vivo*^22^. Here it has been shown that with vacuum packing the membranes along with some desiccant and irradiating them and storing them at low temperatures for 6 months, there is no change in the fiber integrity and degradation. At present, 6 months is as far as we have studied with these membranes containing micropockets but storage data for 1 year has been reported on plain electrospun membranes at -20 °C^22^ and we now have unpublished data for their storage at -20 °C for 2 years without any signs of degradation. Thus for long term storage it would be recommended to store them dry at -20 °C but it is possible to store them at RT even in India for at least 6 months (possibly much longer). The inclusion of a humidity indicator gives an easy means of checking that the packaging has kept membranes dry in which case they will be fit for the purpose.

Transfer of cells from these rings to 3D cornea models was shown when placing limbal explants within the micropockets. This group recently reported transfer of cells onto an *in vitro* rabbit cornea model by placing explants on plain PLGA membranes (membranes without ring structures)^24^. Using the present microfabricated scaffolds cell transfer has been taken one step further as we can now specifically locate tissue explants within the microfeatures. The ability to place the explants directly within the niches also allows the surgeon to use the membranes directly in the surgical theatre avoiding the need of a cleanroom to first expand the limbal stem cells. Although this piece of work has been focused on the development of devices for corneal disease, this microfabrication technology can be also applied for developing devices for many other applications. Future work will explore the fabrication of constructs for the regeneration of other tissues such as skin and bone.

Whilst the design and initial fabrication of the PEGDA microstructures can be time consuming, once fabricated the structures can be reused many times without degradation. Therefore, the subsequent fabrication of PLGA microstructured biodegradable membranes by electrospinning can be performed at a comparable rate to the production of plain ('unstructured') membranes following the assembly of the collector. Although in this work we have used microstereolithography for fabricating the molds, other fabrication methods such as 3D-printing or injection-molding could be also used. Accordingly, the underlying mold could be made out of other polymers or metals instead of PEGDA. As such this technique is very versatile and researchers can easily adapt the method to match their own needs and facilities.

The* in-house* microstereolithography set-up used in this study will not allow the preparation of constructs with features under 30µm; this is not a limitation for the corneal application described here but it could be crucial in the design of other models. In that case other techniques such as 2 photon polymerization (2PP) could be of interest however the electrospinning technique might not allow the reproduction of structures on the sub-micron scale (this is currently being studied by our group).

Critical steps within the fabrication process are (i) Avoiding the overcuring of the PEGDA templates which can be controlled by adjusting time and quantities of photoinitiator. (ii) Controlling electrospinning conditions such as temperature and humidity. (iii) Storing appropriately the electrospun ring membranes using vacuum-packing and desiccants.

In summary, by placing limbal tissue explants within the microfeatures of the membrane we have shown cell outgrowth from the explants on the niche areas, cell transfer onto a rabbit wounded cornea and subsequent re-epithelization of the cornea. The degradation of the membranes stored at different temperatures has also been studied and a packaging protocol which allows long term storage of membranes has been developed, the latter being essential in developing membranes for clinical use.

## Disclosures

The authors have nothing to disclose.
